# Specificity and Areas of Usage of Cardiovascular Prediction Models Among Athletes—State-of-the-art Review

**DOI:** 10.31083/RCM37493

**Published:** 2025-05-16

**Authors:** Tomasz Chomiuk, Przemysław Kasiak, Artur Mamcarz, Daniel Śliż

**Affiliations:** ^1^3rd Department of Internal Medicine and Cardiology, Medical University of Warsaw, 02-091 Warsaw, Poland

**Keywords:** cardiovascular diseases, prediction model, athlete, physical activity, training

## Abstract

Cardiovascular diseases are a leading cause of mortality worldwide. Physical activity is linked with a reduced prevalence of cardiovascular diseases. However, excessive over-volume of training could negatively increase the risk of cardiovascular diseases. Prediction models are usually derived to facilitate decision-making and may be used to precisely adjust the intensity of physical activity and stratify individual exercise capacity. Incorporating prediction models and knowledge of risk factors of cardiovascular diseases allows for the accurate determination of risk groups among athletes. Due to the growing popularity of amateur physical activity, as well as the high demands for professional athletes, taking care of their health and providing precise pre-participation recommendations, return-to-play guidelines or training intensity is a significant challenge for physicians and fitness practitioners. Athletes with confirmed or suspected cardiovascular disease should be guided to perform training in carefully adjusted safe zones. Indirect prediction algorithms are feasible and easy-to-apply methods of individual cardiovascular disease risk estimation. Current knowledge about the usage of clinical forecasting scores among athletic cohorts is limited and numerous controversies emerged. The purpose of this review is to summarize the practical applications of the most common prediction models for maximal oxygen uptake, cardiac arrhythmias, hypertension, atherosclerosis, and cardiomyopathies among athletes. We primarily focused on endurance disciplines with additional insight into strength training. The secondary aim was to discuss their relationships in the context of the clinical management of athletes and highlights key understudied areas for future research.

## 1. Introduction

For many years, research has shown that regular physical activity has a positive effect on health. Reports unequivocally indicate a reduced risk of all-cause mortality, including mortality due to cardiovascular diseases (CVDs) [1]. Physical and endurance training improves metabolism, especially in terms of the respiratory, circulatory, and muscular systems [2]. Although, in general, regular training provides health benefits, it can also have some negative consequences if done unadjusted or at too high of an intensity [3, 4]. The training should be adjusted to individual needs and recovery potential. Among others, the possible methods include psychological measurements, biochemical measurements, and wearable devices [5]. If an athlete, both recreational and professional, does not use any monitoring method, the training could be named as unadjusted [5]. What’s more; some athletes may have previously undiagnosed conditions in the circulatory system or belong to certain risk groups for particular CVDs (primarily amateurs, novices, and elderly athletes).

CVD prevalence and types differ across different age groups [4]. Types and frequencies of CVD that should be considered for advanced young athletes and master athletes are naturally different. Young athletes are usually engaged in more intensive physical training than older senior athletes [6]. Congenital cardiovascular diseases such as hypertrophic cardiomyopathy (HCM), dilated cardiomyopathy (DCM), and many types of coronary anomalies are a priority to discover [7]. On the other hand, for senior athletes, ischemic heart disease is the most important and frequent condition [8].

Recently, many prediction models have been derived to assess the risk of CVD [8]. The original studies are based on different populations, but so far, only a few of them have reported the use of prediction models among athletes [9]. Previous research is often focused only on the variables primarily used to measure exercise performance during cardiopulmonary exercise testing or competition (i.e., heart rate, oxygen uptake, or pulmonary ventilation) [10]. In turn, the clinical models for the prognosis and diagnosis of CVD remain understudied in athletic cohorts. There is a lack of comprehensive summaries for fitness practitioners, sports diagnostic specialists, or medical doctors.

Clinical prediction models are usually divided into prognostic and diagnostic. The first of them is designed to predict the occurrence of the disease in the future, based on the factors available at the time of the test [11]. The diagnostic model aims to calculate the risk that the disease already exists, considering the presented variables [11]. Both types include a plethora of predictors: demographic (age, weight, height), laboratory (serum biomarkers), past medical history, family history, smoking status, dietary habits, and genetics [12]. So far, usually just the existence of risk factors, rather than their crosstalk and relationship, is preferably considered to quantify the risk.

A novel, more individualized approach involves a constant balance between numerous predictors [13]. This flexible procedure enhances the accuracy of forecasted risks and improves the applicability of prediction algorithms for those with multiple diagnosed risk modulators.

**Fig. 1.**
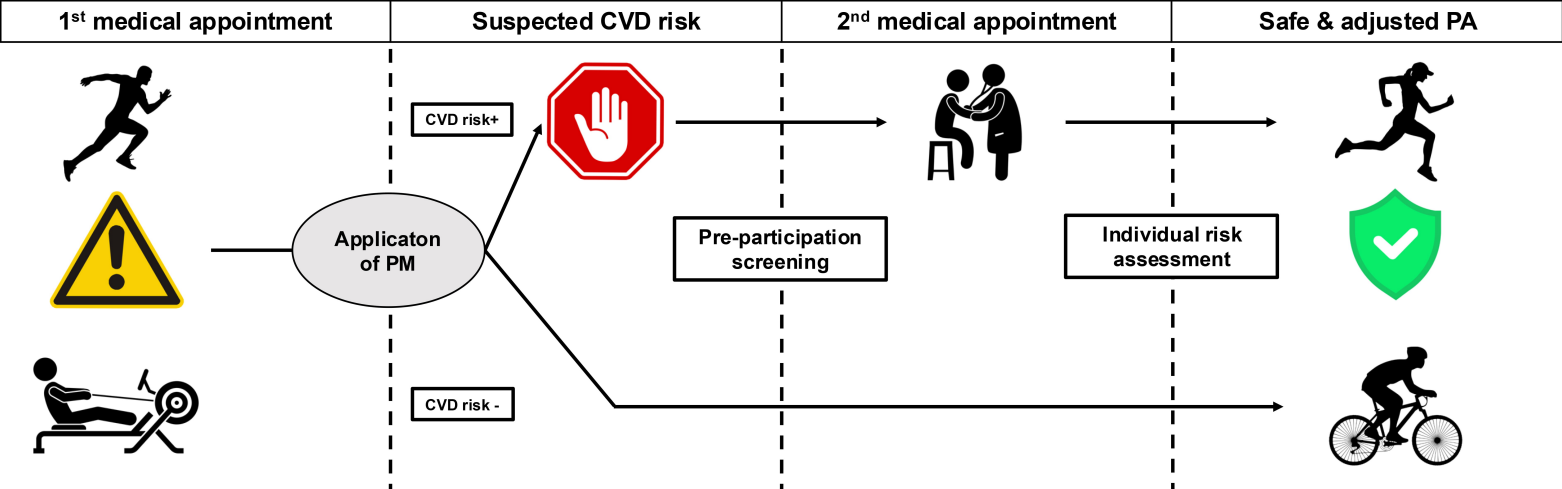
**Central take-home figure**. Proposed protocol of application of prediction models for cardiovascular risk assessment among athletes. Abbreviations: CVD, cardiovascular disease; PA, physical activity; PM, cardiovascular risk prediction model.

A growing number of individuals above 35 years (“master athletes”) and those without training experience (“amateur athletes”), engage in strenuous physical training in various sports disciplines [6]. It warrants increased attention to the weighting between the advantages of exercise and the risk of training-induced negative cardiovascular conditions [14, 15]. Inactive individuals often start practicing endurance disciplines to improve their health and lose weight. Despite being considered healthy, many of them are at the beginning of their path to the burden of CVD risk factors, such as obesity, lipid disorders, hypertension, and diabetes [16]. The personalized risk stratification should provide valuable information to the medical and health professionals (e.g., general practitioner or personal fitness coach) on whether the individual could safely start regular progressive training [14]. Fig. 1 presents the proposed protocol for the use of clinical prediction models for athletes.

Professional athletes can afford regular comprehensive examinations assessing their health status [17, 18]. However, such a complete package of medical testing is usually multistage and expensive. Thus, amateur athletes do not choose it. In consequence, there may be cases of undiagnosed conditions that pose a risk to regular participation in progressive endurance training [17]. The development of a new branch of sports medicine drives the research for alternative solutions. That is the moment when prediction models could be incorporated as a feasible and cost-effective prelude [9]. It is worth underlining that physical activity brings the best health outcomes when it is performed regularly and for a long time. CVD prediction models usually use a forecasting horizon for several years in the future (usually ranging from 2 to 45 years) [8]. They allow preparing guidance in advance for safe and effective long-term physical activity [17]. Both personal coaches and physicians can use such algorithms to properly adjust the intensity and frequency of training sessions.

With the development of numerous prediction models in cardiology, questions arise as to how, when, and which models should be used among athletes. So far, there have been no comprehensive reviews evaluating current knowledge about the practical applicability of CVD prediction algorithms in physically active cohorts. Our goal is to fill this gap. The following article summarizes the latest discoveries in this emerging area and presents them on the background of the most common CVD among athletes. We synthesized epidemiologic data, characteristics of prediction models, and risk factors in clinical cardiology and evaluated their applicability to athletes. Additionally, we outlined understudied areas which require further, more precise research.

## 2. Atrial Fibrillation and Other Minor Arrhythmias

Atrial fibrillation (AF) is described as the chaotic atrial electrical activity that can substitute regular sinus cadence and is one of the most popular dysrhythmias [19]. Usually diagnosed in older adults and individuals at cardiovascular risk (for our purposes — both veteran and amateur athletes), numerous case-controlled research studies have shown its link with a high amount of excessive endurance training [20, 21]. Briefly, excessive training describes physical activity performed above the tolerance level of an individual [22]. The borderline is a subjective measure and depends on the individual’s recovery, life demands, and stressors. An athlete could suspect overtraining when there is no motivation to work, sleep disturbances, elevated resting heart rate, or puissance [22]. Previous studies have noted a high prevalence of AF in middle-aged and master athletes ranging between 12% to 29%. However, despite the high rates of AF, most recommendations focused on other CVDs. It has been argued that AF carries a low risk of serious cardiac events when compared to other CVDs. However, undiagnosed can still lead to some health consequences [15, 21, 23].

Andersen* et al*. [24] illustrated the relationship between AF and overtraining. Skiers who completed ≥5 competitions (hazard ratio (HR) 1.29, 95% confidence interval (CI) 1.04–1.61) and those who had the fastest relative finishing time (HR 1.20; 95% CI 0.93–1.55) were exposed to the highest risk of AF [24]. Despite us knowing the existing relationship between strenuous exercises and AF, underlying mechanisms stay hypothetical and understudied [6]. Numerous preliminary assumptions include raised parasympathetic nervous system activity (so-called “vagal” tone), maladaptively enlarged and pathologically remodeled left atrium, hidden hypertension, inflammation/oxidative stress, periodic cardiac overload, alcohol, and caffeine (which is often supplemented as a performance-enhancing drug) [25, 26]. The accurate dose and threshold of safe physical activity are still debated. We know that low-to-moderate sports did not increase the risk of AF. Although, there is a lack of exactitude for the consequences of chronic, prolonged exposure to over-volume of training demands (beyond which the risk of AF is higher) [6].

The occurrence of AF in the future can be predicted with substantial precision by clinical characteristics. AF risk estimates seem to be a practical go-to method. There are several common prediction scores- CHARGE-AF, C_2_HEST, CHA_2_DS_2_-VASc, EHR-AF, and FHS-AF [27, 28]. According to results from a large comprehensive meta-analysis of 21 AF predictive algorithms, the CHARGE-AF appeared to be most appropriate for preliminary screening, especially when applied to European populations [28]. Among the high heterogeneity of currently available equations, we recommend more unified protocols and cross-validation between different populations of existing models, rather than deriving new ones. This will allow physicians to select the most suitable type for athletes. Screening for the possibility of developing AF and other minor arrhythmias remains important. Cardiac rhythm disorders are common among athletes, primarily in endurance disciplines, and pose some dangers during exercises when undiagnosed [21]. Particularly noteworthy are the more ambitious amateurs who plan to practice high-intensity and high-frequency training and take part in competition events [24].

## 3. Blood Pressure and Hypertension

On average, about 25% of society suffers from elevated blood pressure and this is also mirrored in athletes, both amateur and professional [29]. High blood pressure is more often diagnosed among strength disciplines and bodybuilding. Although it has a lower prevalence among endurance athletes, they are also not excluded from hypertension [29]. It is the most common CVD in athletes and may present some problems with controlling the individual’s eligibility for participating in regular training. Recent reports found an inverse association between endurance sports and blood pressure [30]. Accordingly, physical activity is advised during the treatment to control hypertension [30]. The number of cases of hypertension depends on sport discipline. There are some types of physical activity where the risk may be even higher than in the general population (e.g., powerlifting or bodybuilding) [30]. Undoubtedly, a precise clinical examination is required to make a definitive diagnosis of hypertension among athletes, stratify existing risks, and excluding secondary causalities [31].

In the largest research study of a European cohort, Caselli* et al*. [32] found that hypertension could be found in up to 3% of the athletic population (n = 2040, 64% men). Furthermore, in a wide review (n = 138,390) provided by Berge* et al*. [30], the authors claimed that hypertension prevalence in selected athletic cohorts is comparable to the inactive population. Although, results depend on the kind of discipline, and hypertension is more often seen in strength than endurance disciplines. Potential hypertension-predisposing risk factors are higher body mass index (often seen in amateurs and novices or bodybuilders with high muscle mass) and chronic misuse of illegal drugs or painkillers (predominantly found among professionals) [32]. Irregular circadian rhythm during competition season could also contribute to elevated blood pressure [32]. Tournaments or races during different phases of the day and on different continents could lead to sleep disorders which are related to hypertension [33]. Additional influencing variables may be a family history and genetic variables [34].

Thus, risk forecasting may be important in screening individuals with the highest possibility of current hypertension or developing it in the future [35]. Precise knowledge helps physicians select those who will benefit the most from regular medical follow-up [35]. Prediction models for hypertension are one of the most numerous in clinical cardiology [8]. The universal and thoroughly validated algorithm is the Framingham Risk Score and its derivatives [8, 35, 36, 37, 38, 39]. Analyzing data from the Framingham cohort study, the researchers derived an uncomplicated scale with fair performance. The proposed algorithm classifies individuals into low (5%), medium (5% to 10%), or high (10%) likelihood of suffering hypertension in the following four years [35]. Calculations are based on points given for age, sex, blood pressure (both systolic and diastolic), body mass index, smoking status, and family history. Similar risk factors are common among other CVD, and athletes should be reassured about controlling them [8].

There are also regression equations to predict blood pressure variability during graded exercise. Two of them are provided by Mascherini* et al*. [40] and Szmigielska* et al*. [41]. Considering resting systolic and diastolic blood pressures, body mass index, age, and sex, it is possible to estimate peak blood pressure during workload. Such data and their comparison with resting measurements allows physicians and coaches to find those with abnormal blood pressure responses and refer them to further clinical evaluation [42].

To sum up, healthcare practitioners can utilize the Framingham scores or exercise regression equations to estimate an individual’s chances of hypertension and monitor cardiac stress response [40]. Further steps are to inform the athletes of potential risks and assist during the choice of sport, threshold, amount and duration of physical activity. In addition, the proposed method of screening is relatively inexpensive, widely accessible, and uncomplicated. It is a viable tool for fitness practitioners to refer an athlete with suspected abnormal blood pressure to a medical appointment. This approach helps find so-called “red flags”, i.e., athletes requiring precise intensity monitoring to safely participate in graded, progressive training. Furthermore, we recommend deriving new formulas with novel predictors of hypertension among athletes. Additional risk factors often found in sported cohorts are sleep assessments and primary sport discipline. Perhaps, their assessment could be completed using a questionnaire. New algorithms profiled for a specific population will be more useful for the medical team and coaching staff.

## 4. Coronary Artery Disease

Coronary artery disease (CAD) is one of the most common CVDs in the developed world and is associated with high morbidity [43]. Undoubtedly, endurance training reduces the risk of CAD [44]. However, current research has indicated a paradoxical association between excessive levels of conditioning and elevated CAD prevalence [45]. The underlying mechanism remains unclear, but existing findings documented raised serum levels of parathyroid hormone and consequently higher blood Ca^2+^ after workout bouts. It may explain to some degree the increased incidence of CAD [46, 47].

In the general population, 2.4% to 6.6% of regular, symptomatic patients need noninvasive screening for possible high-risk CAD [48]. A simple set of preclinical factors enhances the prediction performance of CAD over the standard clinical examination. The identification of the highest-risk groups may facilitate decision-making related to additional tests, catheterization, or medical therapy [48]. For the athletic population, such a protocol may include adjusting and decreasing intensity to safe zones.

In a cross-sectional study on middle-aged and master athletes, Aengevaeren* et al*. [49] linked the occurrence of atherosclerotic plaques and elevated coronary artery calcification with prolonged, excessive conditioning. Training intensity but not amount, was correlated with aggravation of CAD in a six-year horizon. Strenuous training was responsible for greater CAD incidence and calcified plaque development. Whereas low-intensity exercise resulted in reduced CAD progression. Findings suggest that it is worth paying attention to the intensity prescribed to athletes and cardiac patients at a high risk of developing CAD [49].

Recently, Jang* et al*. [48] provided two prediction models of CAD. The algorithms consisted of practical, easy-to-obtain factors accessible from patients’ medical history. There were seven variables, although particular importance in a sport-setting should be placed on family history, advanced age, and male sex. CAD risk factors correspond well with those described above for other CVDs [16]. Amateurs who start regular exercise and elderly veterans may suffer from them. Jang* et al*. [48] underlined that direct medical screening for individuals at high risk of CAD may be problematic. Hence, we shouldn’t leave athletes unattended. Similar conclusions were provided by Hwang* et al*. [50]. Their findings confirmed results from previous studies. General practitioners and sports medicine physicians should be acknowledged with cohorts mostly exposed to CAD development to properly allow sports participation. Special attention should be paid to endurance disciplines among amateurs and the elderly.

To sum up, Lenselink* et al*. [51] examined the accuracy and transferability of 28 CAD prediction models on independent wide samples. Despite some inaccuracies, current algorithms are well-calibrated and replicable. They can be used by healthcare practitioners when conducting a comprehensive pre-participating medical evaluation or prescribing return-to-play guidelines [51].

## 5. Cardiomyopathies 

Strenuous endurance conditioning can induce a specific pattern of functional and anatomical adaptations in the circulation [52]. In an unspecified ratio, an Athlete’s Heart occurs. Precise diagnosis could strongly contribute to the prevention of particular athletes from sudden cardiac death [52]. However, those with an Athlete’s Heart may continue their careers, even as competitors [52]. Recent reports related to the course of cardiac hypertrophy suggest that an individual approach may be the best management strategy [52]. Athlete’s Heart results from biventricular hypertrophy [53]. There are also conditions with similar etiology, e.g., arrhythmogenic right ventricular cardiomyopathy (ARVC) [24]. Briefly, ARVC is a genetically acquired cardiomyopathy influencing tissue desmosomes and is described by the fibro-fatty substitute of typical cardiac myocytes, predominantly in the structure of the right ventricle [24].

Existing data suggest that endurance exercise accelerates the penetrance of the ARVC phenotype in those who are confirmed to carry the gene, consequently hurrying the evolution of severe ventricular arrhythmias and heart failure [54]. It has been stipulated that numerous episodes of exercise-induced growth in pulmonary pressures may contribute to stronger afterload for the right ventricle and promote pathologic adaptation and cardiac remodeling which results in ARVC [24]. Currently, numerous unanswered queries and knowledge gaps have emerged. In the majority, conclusions are preliminary and based on observational studies from small populations [6]. Despite speculative mechanisms, undiagnosed myocardial fibrosis can occur in veterans after finishing a competitive career [6]. This condition results most likely from external factors, disconnected with training experience (perhaps post-career inappropriate lifestyle habits, alcohol consumption, unhealthy dietary preferences, periodical drug abuse, and performance-enhancing drugs during competitive times) [6]. It is worth noting that veterans or masters and amateurs are especially exposed to them.

A new risk prediction protocol for ARVC has been developed by Chen* et al*. [55] on an international cohort of 389 patients. Researchers analyzed popular clinical parameters such as left ventricular ejection fraction, serum creatinine levels, tricuspid regurgitation, and AF [55]. All of them could select athletes who are in danger of terminal events. They may advise physicians to consider and optimize follow-up procedures and rehabilitation exercise programs. This new prediction model also indicates the need for registering high-risk individuals and those with confirmed ARVC on the appropriate waiting lists [55].

The current prediction models mostly focus on ARVC. However, the HCM and DCM could also occur among athletes and are a serious issue. HCM could lead to fatal cases and is one of the most common cases of sudden cardiac death among athletes [7]. The diagnosis of HCM in physically active populations could not be easy and differential diagnosis is complex [7]. DCM could be similar to Athlete’s Heart and prediction models could facilitate differential diagnosis [56]. The formulae could be considered the most common sign among athletes. For example, covariates for such a model could include ejection fraction, electrocardiography (ECG), echocardiography results, etc. Such a tool in a comprehensive diagnostic process could discriminate DCM and physiological left ventricular dilation in athletes [56]. Therefore, further prediction models could also be derived to stratify the risk of HCM and DCM.

Athlete’s Heart and other cardiomyopathies are widely described syndromes [53]. However there is a lack of risk forecasting guidelines for safe exercises in those conditions. We recommend deriving novel prediction algorithms, perhaps including advanced contributing factors from radiological imaging and genetic testing.

## 6. Other CVDs

There are numerous prediction models derived for remaining the CVDs [8]. Among others, they concern stroke, myocardial infarction, or survival after invasive procedures [9]. It is worth highlighting that the majority of models are based on a common and replicable set of predictors [8, 16]. Basic, universal CVD risk factors include nutrition quality, physical activity, nicotine exposure, sleep, body mass index, blood pressure, blood glucose, and lipid metabolism. The better the scores, the lower the risk of dying from CVD and all-cause mortality [57]. Actions that encourage optimal scores should be promoted among the athletes regardless of the level of advancement and age. Medical professionals and fitness practitioners should acknowledge the most common contributing variables to properly prescribe pre-participation guidelines and return-to-play protocols or adjust training. This is a universal tip to optimize results in other CVD risk estimations.

## 7. Maximal Oxygen Uptake (VO_2max_) and its Role in Predicting CVD

VO_2max_ is a thoroughly described indicator of cardiovascular fitness and endurance capacity [58]. People with higher VO_2max_ have lower all-cause mortality, especially related to CVD [59]. Years of research in cardiology, epidemiology, and sports diagnostics have determined that a higher VO_2max_ is linked with a multitude of health benefits [60]. The impact of low cardiorespiratory fitness on cardiovascular and all-cause mortality is stronger than other predictors of CVD [61]. VO_2max_ is a parameter that merges the function of respiratory, muscular, and circulatory systems and gives an outlook of overall body physical performance [58]. Endurance training enables us to improve and maintain a high stable VO_2max_ with age [61]. Individuals who do not exercise regularly, experience a steeper VO_2max_ decline compared to active individuals [62]. In consequence, high VO_2max_ prevents CVD occurrence [61].

The gold standard for measuring VO_2max_ is the maximal symptom-limited cardiopulmonary exercise test [63]. However, due to practical reasons, such as lack of specialized testing equipment or personnel and the costs of the procedure [64], it is often infeasible to conduct studies on wider populations.

Hence, various equations for indirect VO_2max_ calculation have been derived. There are a significant number of VO_2max_ prediction models. We can classify them into linear regression models, which predict VO_2max_ level based on somatic and exercise variables [62], and prognostic-diagnostic models, which forecast cardiovascular events and mortality based on VO_2max_ [65].

Their accuracy is assessed in validation studies [62, 66, 67]. VO_2max_ prediction models directed for all-cause and CVD mortality could be used as a valuable alternative to direct measurement, especially when recalibrated for the target population [62, 68, 69].

In recent years, there has been an emerging role of other cardiorespiratory parameters in predicting and diagnosing CVD. In particular, the oxygen uptake efficiency slope (OUES), oxygen uptake efficiency plateau (OUEP), ventilatory efficiency (VE/VCO_2_), and peak oxygen pulse (O_2_P_peak_) gained attention [70, 71, 72, 73]. The comprehensive role of VO_2max_ and its interaction with other parameters is crucial to understanding the risk of CVD, especially in narrow and specific populations [59]. For example, higher VO_2max_ indicates a lower risk of CVD, but higher VE/VCO_2_ suggests worse cardiorespiratory fitness [74]. OUES and OUEP are more attractive during the cardiopulmonary exercise test (CPET) because both do not require maximal effort to be derived [75]. Finally, O_2_P_peak_ most precisely mirrors the function of the left ventricle responsible for ejection fraction, and is therefore a key measure for endurance athletes [76].

There is a lack of consensus on the discernable set of universal covariates for scaling cardiorespiratory fitness. Available models are often based on different predictors. This makes direct comparisons problematic. When choosing a predictive equation, determining characteristics should evaluate the precise level of exertion, previous medical history, drug history, derivation, validation cohorts, and testing modality [66]. Correct application of predicted VO_2max_ to stratify endurance capacity or CVD risk is a valuable method of guiding with precisely adjusted intensity for fitness training and medical rehabilitation [58].

Despite the unparalleled impact of VO_2max_ (both measured or estimated) on CVD prevalence, all the above-discussed CVD prediction models did not include it as a covariate. We recommend adding VO_2max_ and other indicators of cardiorespiratory fitness in future clinical prediction models to improve their predictive accuracy.

## 8. Discussion

This review examines the utility of CVD prediction models in athletes, emphasizing the need for individualized risk assessments in physically active populations. While regular exercise reduces CVD risk, excessive training can pose cardiovascular threats, particularly in amateur, veteran, and professional athletes. The study highlights gaps in research, such as the underuse of prediction models in athletic cohorts, and proposes protocols for integrating these tools into pre-participation screening. Specific CVDs are prevalent among athletes, including AF, hypertension, and cardiomyopathies, are discussed, alongside the role of VO_2max_ and other cardiorespiratory measures in risk prediction. Future directions include novel predictors, external validation, and ethnic adaptations. Overall, prediction models offer a cost-effective tool for preliminary CVD risk stratification, aiding safe training practices.

## 9. Challenges in Applying Prediction Models to Athletes

Currently, prediction models forecast the risk or diagnosis of CVDs and where these are likely to be fatal or non-fatal [8]. However, there are still emerging areas to discover. In clinical settings, numerous, already known variables are linked with CVD (e.g., albuminuria, education level, and coronary artery calcium). Despite having a confirmed predicting value, they are not employed to build more precise prediction algorithms. Perhaps the flexible incorporation of novel risk modeling factors will enrich the value of forecasting several clinically relevant cardiac conditions [77]. In addition, the proposed novel direction is to supplement existing formulas with cardiorespiratory fitness indicators, perhaps VO_2max_.

The final decision of whether an athlete can be involved in training and competing is of crucial importance [78]. Prediction models cannot be used to draw a final diagnosis but could help during a comprehensive examination process and indicate the individuals at the highest risk [9]. This issue also depends on whether the decisive person is a medical doctor or not (e.g., a coach or a sports scientist) [78]. This is where the emerging role of shared decision-making allows avoidance of accidents during training or games and simultaneously prevents the unnecessary exclusion of an athlete from sport [78].

The preferred sports discipline is also a key factor when applying the prediction models as CVD risks may vary significantly across different sport disciplines [79]. For example, strength athletes more often suffer from hypertension than endurance athletes [29]. Moreover, endurance athletes have a higher risk of sarcopenia with aging, compared tostrength athletes [80]. The protective impact on the cardiovascular system of exercises is most often attributed for endurance sports (running, cycling, etc.) [81]. The cardiac effects of detraining are mostly visible in endurance disciplines rather than strength sports [81]. Moreover, the usage of doping substances that could aggravate the CVD risk is more common among strength sports [82]. Moreover, traditional risk factors could be subtle or temporary among athletes. For example, biochemical markers could grow due to strenuous training and some lipid disorders could even occur in strength athletes during dieting [83]. Finally, all the differences between types of sports or athletes and the general population should be acknowledged. Sports discipline should be considered when applying any diagnostic protocol.

Many of the above-discussed prediction models have not yet been externally validated. The most frequently examined models for general populations are: Framingham, SCORE, and QRISK [8, 35, 84, 85]. It would be interesting to see their head-to-head performance among protocols in similar fields and direct validations on athletic samples. Moreover, studies most often include patients from Europe and North America [8, 86]. However, athletes are all ethnicities. Body physiology and exercise capacity vary slightly between ethnic groups [87, 88]. Thus, seeing the performance of the prediction formulae on other populations, e.g., African, Australian, or South American, would give valuable insights. The actual risk stratification approach suggests that the goal is to generate a clinically pertinent CVD prediction model and additionally combine current algorithms [8].

In particular, the criteria for participation in exercise with near-all-out intensity in seniors include factors which are often difficult to judge [89]. Master athletes should precisely monitor their efforts and recovery [5]. Any worrying signs such as breathlessness or chest pain should be the termination points of exercises [62]. Moreover, senior athletes must acknowledge the decrease in endurance capacity with aging (e.g., VO_2max_ decrease of about 6–8 mL/kg/min per decade) [60]. Older athletes should use monitoring methods and technologies, like wearables, to avoid overtraining [90]. Finally, regular medical follow-up supported by precise cardiopulmonary assessment (e.g., via CPET) could facilitate safe sport [59].

In Fig. 1 we proposed a protocol consisting of four phases. The first phase is illustrated in the left-lateral box. At this stage, CVD risk is unknown. A physician or fitness practitioner makes a preliminary CVD risk assessment based on estimation via prediction models. The second and third phases are illustrated in the central boxes (left and right respectively). If CVD is suspected (i.e., CVD risk +), the athlete is referred for precise medical evaluation. Additional diagnostic steps may include a medical appointment with a specialist, laboratory tests, cardiopulmonary exercise tests, etc. to make a definitive diagnosis. In the absence of suspicion of CVD (i.e., CVD risk –), the athlete is referred to basic diagnostic tests and regular health monitoring. The fourth step is illustrated in the right-lateral box. The process ends with approval for athletes with safe, adjusted, and precisely monitored physical activity (PA). The presented protocol enables feasible, inexpensive, and widely accessible forms of preliminary medical screening, in particular CVD risk assessment among athletes. Its usage enriches athletes’ adherence to pre-participation screening and, in consequence, raises their diagnostic potential. It does not exempt athletes from periodic medical evaluation and health monitoring. However, it is an initiatory stage that facilitates the selection of athletes requiring deep, precise health assessment.

**Fig. 2.**
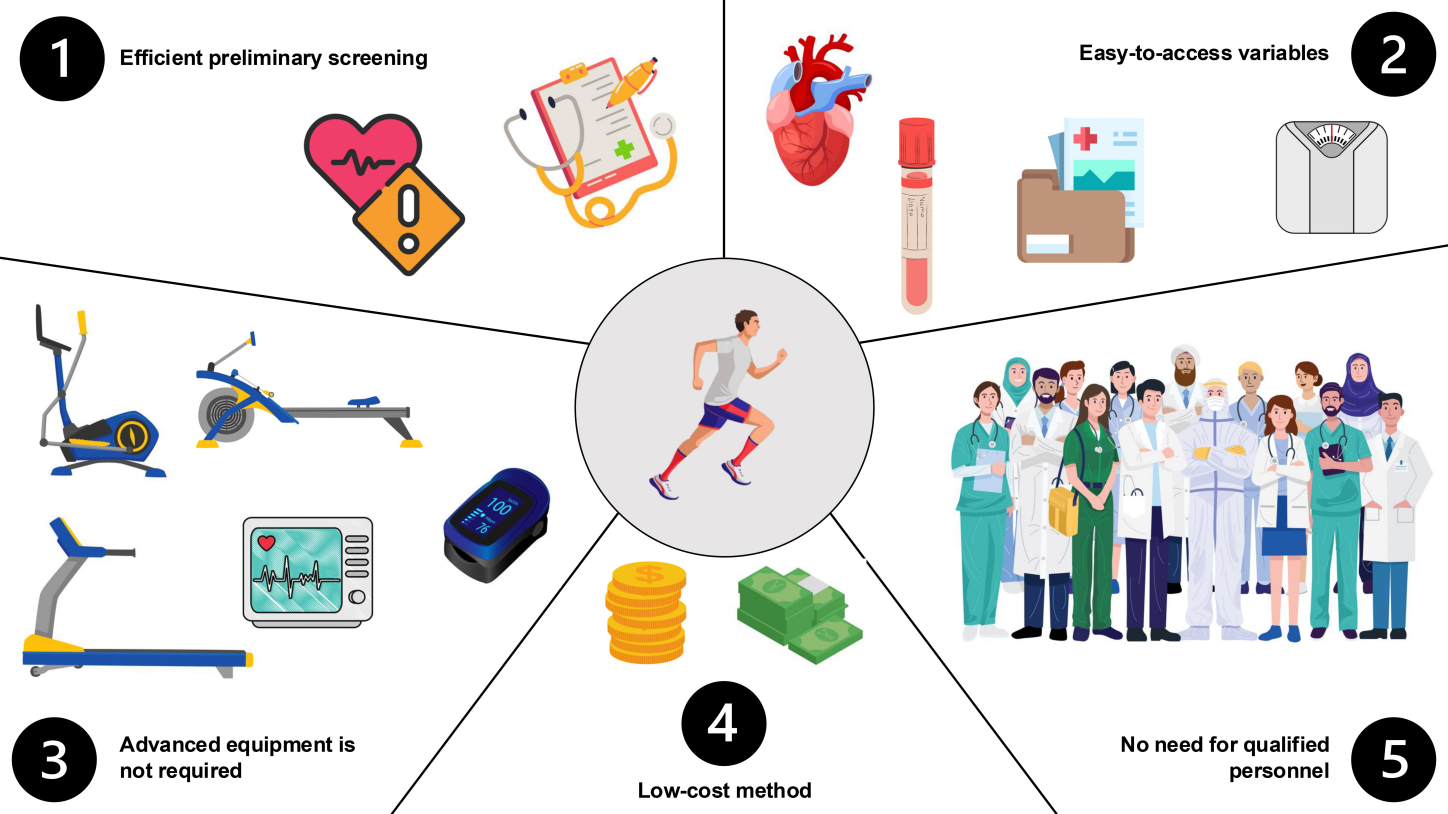
**Main advantages of using prediction models among athletes**.

In Fig. 2, we illustrated the most important and emerging advantages of predictive models in cardiovascular risk stratification. Such algorithms provide a feasible and practical approach for physicians and fitness practitioners when guiding physically active individuals. (1) Early identification of increased risk of cardiovascular events during pre-participation screening is facilitated. Athletes with suspected elevated risk could be guided for a further, more comprehensive health assessment. (2) Most algorithms include widely available variables such as anthropometric measurements, demographic characteristics, or basic medical tests. Therefore, there is no need to undergo a complex medical examination if the preliminary risk is low or during periodic, regular health assessments. (3) Current prediction models limit the necessity to use specialized fitness equipment or medical devices. Initial risk stratification could be conducted during a brief appointment or by telemedicine advice. Subsequently, if an elevated risk is suspected, the athlete may be recommended to undergo a full diagnostic protocol. (4) There is no need to conduct a full exercise protocol, extensive laboratory examinations, etc. Thus, prediction models allow for higher compliance due to the simplification and reduction in costs to obtain basic pre-participation approval. As most athletes have to pay for medical appointments out of their own finances, it is one of the main reasons for non-adherence to medical screening. (5) The health assessment can be performed without the involvement of numerous medical staff (e.g., nurse, physician, lab technician, exercise physiologist). Thus, medical personnel can devote more time to individuals with severe health conditions.

Undoubtedly, the gold standard would always be to conduct a full screening and diagnostic examination of each athlete. In practical circumstances, this is difficult to implement due to the costs of the procedures, their complexity, and the availability of diagnostic centers. This is the moment when prediction models come for a preliminary screening assessment. They provide a cost-effective prelude to a thorough medical examination [9]. They help to find so-called “red flags”, i.e., athletes who are most likely to develop certain diseases or have the highest risk of an ongoing condition. Those should be referred for more detailed diagnostic tests to ensure safe and effective training.

In Table 1 (Ref. [36, 37, 38, 39, 40, 41, 50, 55, 84, 85, 91, 92, 93, 94, 95, 96, 97, 98, 99, 100, 101, 102]) we described the most common categories of CVD risk factors among athletes and indicated their type. Additionally, we propose major exposure groups with the highest probability where selected risk factors could occur. We provided the most often validated (>3 external validations) prediction models and prediction models described in this review which include particular variables in risk stratification. Three subgroups of athletes were proposed to facilitate risk stratification: (1) ‘amateurs and new’ refers to athletes with limited sports experience and those who started endurance training to improve their health who previously were inactive with an unhealthy lifestyle, (2) ‘veterans and masters’ refers to people who finished their competitive sports career or are amateurs at an advanced age, and (3) ‘professionals’ refers to individuals who competitively take part in sports events, are exposed to significant training demands and possibly use illicit performance-enhancing drugs.

**Table 1.** **Characteristics of cardiovascular risk factors among athletes**.

Risk factor category	Predictor	Type	Primary exposed group	Prediction model
Demographics and origin	Age	Non-modifiable	Veterans and masters athletes	∙ Framingham Anderson [37]
				∙ Framingham Wilson [91]
				∙ SCORE [85]
				∙ Framingham D’Agostino [36]
				∙ Framingham ATP III [38]
				∙ QRISK [84]
				∙ PROCAM [102]
				∙ Framingham Wolf [39]
				∙ Friedland* et al*. [92]
				∙ Keys* et al*. [93]
				∙ ASSIGN [94]
				∙ Chien* et al*. [95]
				∙ Asia Pacific cohort studies [96]
				∙ Framingham Risk Score [97]
				∙ Szmigielska* et al*. [41]
				∙ Mascherini* et al*. [40]
				∙ Hwang* et al*. [50]
				∙ Jang* et al*. [98]
				∙ CHARGE-AF [99]
	Sex		∙ Veterans and masters athletes	∙ Framingham Anderson [37]
			∙ Amateurs and new athletes	∙ Framingham Wilson [91]
			∙ Professional athletes	∙ SCORE
				∙ Framingham D’Agostino [36]
				∙ Framingham ATP III [38]
				∙ QRISK
				∙ ASSIGN
				∙ Chien* et al*. [95]
				∙ Asia Pacific cohort studies [96]
				∙ Szmigielska* et al*. [41]
				∙ Jang* et al*. [98]
				∙ CHARGE-AF
	Family history			∙ PROCAM
				∙ Framingham Risk Score
				∙ Hwang* et al*. [50]
				∙ Jang* et al*. [98]
	Ethnicity			∙ QRISK
				∙ CHARGE-AF
	Genetics			None
Comorbidities	Diabetes and glucose intolerance	Modifiable	Amateurs and new athletes	∙ Framingham Anderson [37]
				∙ Framingham D’Agostino [36]
				∙ QRISK
				∙ PROCAM
				∙ Chambless* et al*. [100]
				∙ Friedland* et al*. [92]
				∙ ASSIGN
				∙ Hwang* et al*. [50]
				∙ Jang* et al*. [98]
				∙ CHARGE-AF
	Atrial fibrillation		Professional athletes	∙ QRISK
				∙ Framingham Wolf [39]
				∙ Chen* et al*. [55]
	Past medical history		∙ Veterans and masters athletes	QRISK
			∙ Amateurs and new athletes	
			∙ Professional athletes	
Lifestyle	BMI		Amateurs and new athletes	∙ QRISK
				∙ Chambless* et al*. [100]
				∙ Keys* et al*. [93]
				∙ Chien* et al*. [95]
				∙ Framingham Risk Score
				∙ Mascherini* et al*. [40]
				∙ CHARGE-AF
	Smoking			∙ Framingham Anderson [37]
				∙ Framingham Wilson [91]
				∙ SCORE
				∙ Framingham D’Agostino [36]
				∙ Framingham ATP III [38]
				∙ QRISK
				∙ PROCAM
				∙ Chambless* et al*. [100]
				∙ Friedland* et al*. [92]
				∙ Keys* et al*. [93]
				∙ Leaverton* et al*. [101]
				∙ ASSIGN
				∙ Chien* et al*. [95]
				∙ Asia Pacific cohort studies [96]
				∙ Framingham Risk Score
				∙ CHARGE-AF
	Stress and socioeconomic status			ASSIGN
	Diet			None
	Alcohol			None
Blood lipids and serum biomarkers	Total cholesterol		∙ Veterans and masters athletes	∙ Framingham Anderson [37]
			∙ Amateurs and new athletes	∙ Framingham Wilson [91]
				∙ SCORE
				∙ Framingham D’Agostino [36]
				∙ Framingham ATP III [38]
				∙ Chambless* et al*. [100]
				∙ Friedland* et al*. [92]
				∙ Keys* et al*. [93]
				∙ Leaverton* et al*. [101]
				∙ ASSIGN
				∙ Chien* et al*. [95]
				∙ Asia Pacific cohort studies [96]
	HDL cholesterol			∙ Framingham Anderson [37]
				∙ Framingham Wilson [91]
				∙ Framingham D’Agostino [36]
				∙ Framingham ATP III [38]
				∙ PROCAM
				∙ Chambless* et al*. [100]
				∙ ASSIGN
				∙ Chien* et al*. [95]
	Non-HDL cholesterol			None
	LDL cholesterol			∙ PROCAM
				∙ Chien* et al*. [95]
BP	Systolic BP		∙ Veterans and masters athletes	∙ Framingham Anderson [37]
			∙ Amateurs and new athletes	∙ Framingham Wilson [91]
				∙ Framingham D’Agostino [36]
				∙ Framingham ATP III [38]
				∙ PROCAM
				∙ Chambless* et al*. [100]
				∙ Keys* et al*. [93]
				∙ Leaverton* et al*. [101]
				∙ Chien* et al*. [95]
				∙ Asia Pacific cohort studies [96]
				∙ Framingham Risk Score [97]
				∙ Szmigielska* et al*. [41]
				∙ CHARGE-AF
	Diastolic BP			∙ Framingham Anderson [37]
				∙ Mascherini* et al*. [40]
				∙ CHARGE-AF
	Hypertension and BP			∙ Framingham Wilson [91]
				∙ SCORE
				∙ Framingham ATP III [38]
				∙ QRISK
				∙ Chambless* et al*. [100]
				∙ Framingham Wolf [39]
				∙ Friedland* et al*. [92]
				∙ ASSIGN
				∙ Hwang* et al*. [50]
				∙ Jang* et al*. [98]
				∙ CHARGE-AF

Abbreviations: BMI, body mass index; HDL, high-density lipoprotein; LDL, low-density lipoprotein; BP, blood pressure.

## 10. Conclusions

Given the growing popularity of endurance disciplines and the increased number of amateur, veteran, and professional athletes, there is a need for individualized diagnostic and screening approaches. To prevent harmful, unforeseen effects of CVD, a promising method is provided by using prediction models. Existing equations require evaluation of transferability and should be adjusted for the specificity of the athletic cohorts. It must be highlighted that currently, such algorithms can only be a supplemental method despite promising results on general populations. Medical professionals and fitness practitioners could apply indirect predictions during screening, but these are not for definitive diagnoses or to prevent physical activity among athletes.
